# Prevalence of tick-borne bacterial pathogens in Germany—has the situation changed after a decade?

**DOI:** 10.3389/fcimb.2024.1429667

**Published:** 2024-07-18

**Authors:** Katja Mertens-Scholz, Bernd Hoffmann, Jörn M. Gethmann, Hanka Brangsch, Mathias W. Pletz, Christine Klaus

**Affiliations:** ^1^ Institute of Bacterial Infections and Zoonoses, Friedrich-Loeffler-Institut – Federal Research Institute for Animal Health (FLI), Jena, Germany; ^2^ Institute of Infectious Diseases and Infection Control, Jena University Hospital, Jena, Germany; ^3^ Institute of Diagnostic Virology, Friedrich-Loeffler-Institut – Federal Research Institute for Animal Health (FLI), Greifswald-Insel Riems, Germany; ^4^ Institute of Epidemiology, Friedrich-Loeffler-Institut – Federal Research Institute for Animal Health (FLI), Greifswald-Insel Riems, Germany

**Keywords:** *Borreliella* spp., *Rickettsia* spp., *Anaplasma phagocytophilum*, *Coxiella burnetii*, *Ixodes ricinus*, prevalence

## Abstract

**Introduction:**

Tick-borne pathogens, such as *Borreliella* spp., *Rickettsia* spp., and *Anaplasma* spp., are frequently detected in Germany. They circulate between animals and tick vectors and can cause mild to severe diseases in humans. Knowledge about distribution and prevalence of these pathogens over time is important for risk assessment of human and animal health.

**Methods:**

*Ixodes ricinus* nymphs were collected at different locations in 2009/2010 and 2019 in Germany and analyzed for tick-borne pathogens by real-time PCR and sequencing.

**Results:**

*Borreliella* spp. were detected with a prevalence of 11.96% in 2009/2010 and 13.10% in 2019 with *B. afzelii* and *B. garinii* as dominant species. *Borrelia miyamotoi* was detected in seven ticks and in coinfection with *B. afzelii* or *B. garinii*. *Rickettsia* spp. showed a prevalence of 8.82% in 2009/2010 and 1.68% in 2019 with the exclusive detection of *R. helvetica*. The prevalence of *Anaplasma* spp. was 1.00% in 2009/2010 and 7.01% in 2019. A*. phagocytophilum* was detected in seven tick samples. None of the nymphs were positive for *C. burnetii*.

**Discussion:**

Here, observed changes in prevalence were not significant after a decade but require longitudinal observations including parameters like host species and density, climatic factors to improve our understanding of tick-borne diseases.

## 1 Introduction

Tick-borne diseases represent a severe medical problem in many regions. In Europe, Lyme borreliosis (LB) and tick-borne encephalitis are of special importance with very high incidence rates of >100/100,000 population or >15/100,000 in some European countries (Burn et al., 2023; Van Heuverswyn et al., 2023). Furthermore, other bacterial, viral, and protozoal pathogens can be transmitted by ticks and cause diseases in humans and animals. In Europe, these infections are mainly transmitted by the hard tick *Ixodes* (*I*.) *ricinus*. This tick species has a wide host spectrum, and tick activity can be observed in association with a broad range of biotic and abiotic factors, for example, between 3°C and 28°C air temperature and between 35% and 95% air humidity (Gethmann et al., 2020).

Rising temperatures (climate change), land management, and acaricide resistance are possible drivers of the spread of ticks into new territories or increased tick populations (Nuttall, 2022; Vanwambeke et al., 2024). These ticks may carry new pathogens and represent a financial burden to the public health system and veterinary public health system. Therefore, monitoring of ticks and tick-borne pathogens is essential to understand transmission dynamics, to raise awareness, and to adjust counter measures (treatments, vaccines, and acaricides) (Harrington et al., 2020). It has been estimated from available epidemiological data of some federal states and national health insurances that LB constitutes between 60,000 and >200,000 human infections in Germany every year (Wilking et al., 2023). The *Borrelia burgdorferi* sensu lato (s.l.) group, recently phylogenetically reclassified as *Borreliella* (*B*.) spp., includes at least 20 genospecies, and three further genospecies were under discussion (Gupta, 2019; Wolcott et al., 2021). According to present knowledge, only infections with *B. burgdorferi* sensu stricto (s.s.), *B. garinii*, *B. afzelii*, *B. spielmanii*, and, since 2009, *B. bavariensis* (former *B. garinii* OspA-type 4) cause clinical symptoms in humans (Margos et al., 2009; Rauer et al., 2018).

*Borrelia miyamotoi*, genetically more closely related to *B. burgdorferi* s.l. than to the relapsing fever (RF) spirochetal group, was first detected in 1994 in ticks in Japan. Now, it occurs in the northern hemisphere and co-circulates with *B. burgdorferi* s.l., the causative agent of LB with overlapping vertebrate and tick hosts. Human cases caused by *Borrelia miyamotoi* are rare and mostly present as influenza-like illness, with RF in sporadic cases (Cutler et al., 2019).

Another group of emerging tick-borne pathogens are bacteria of the order *Rickettsiales*. They are widespread, obligate intracellular and host-adapted bacteria. Among other genera of the order *Rickettsia* and *Anaplasma* are of human public health and agricultural interest. They are maintained between the animal host and tick vector.

*Rickettsia* spp. cause febrile illness of varying severity in humans. The bacteria are maintained in small mammals, e.g., rodents and the tick vector. Humans are accidental hosts where the bacteria preferentially replicate within endothelial cells of blood vessels causing vascular inflammation and a typical rash or eschar at the side of inoculation. If untreated, the infection may progress to meningoencephalitis and multiorgan failure (Helminiak et al., 2022). Most prevalent species in Northern Europe and Germany belong to the spotted fever group (SFG) rickettsiae, such as *R. helvetica* and *R. monacensis* (Scheid et al., 2016; Guccione et al., 2023). The pathogenic potential of *R. helvetica* varies from mild to severe and may progress to chronic perimyocarditis and meningitis (Scarpulla et al., 2018). The pathogenicity of *R. monacensis* is unknown, and clinical cases are rarely described (Jado et al., 2007). Both species are transmitted by *I. ricinus*, and a high prevalence has been reported for *R. helvetica* in ticks with up to 12% in Germany (Wolfel et al., 2006).

*Anaplasma* can affect animals as well as humans and replicate within cells of the hematopoietic system. The distribution is closely related to the tick vector with domestic and wild animals as reservoir. Three *Anaplasma* species have been reported in Europe, whereof *A. phagocytophilum* is the only species with a high zoonotic potential (Hofmann-Lehmann et al., 2004; Bauer et al., 2021; Knoll et al., 2021b). It is the etiological agent of human granulocytic anaplasmosis (HGA), and clinical symptoms vary from mild to severe febrile illness with headache, malaise, and myalgia (MacQueen and Centellas, 2022). Domestic animals such as dogs, cats, and horses may develop granulocytic anaplasmosis with various clinical symptoms (Hofmann-Lehmann et al., 2004; Jensen et al., 2007; Schafer et al., 2022b). In cattle and sheep, an infection with *A. phagocytophilum* causes tick-borne fever indicated by high fever, anorexia, and signs of depression. In cattle, a decreased milk yield and abortions are observed (Woldehiwet, 2006). *A. phagocytophilum* is transmitted by *I. ricinus*, the main vector in Europe, whereas *Dermacentor* spp. ticks seem to play a lesser role. The prevalence in vertebrate hosts varies among European countries with up to 34% (Stuen et al., 2013).

*Coxiella burnetii* is the etiological agent of Q (query) fever causing acute flu-like illness in humans, which can become chronic and life-threatening. It is endemic worldwide except New Zealand. It has a very broad host spectrum, and domestic ruminants are considered as the main reservoir. *C. burnetii* was originally isolated from a *D. andersoni* tick in Montana, USA, and, since then, the role of ticks in transmission of *C. burnetii* is controversially discussed. Especially, with the discovery of *Coxiella*-like endosymbionts (CLEs), the relevance of ticks in the epidemiology of Q fever is debatable (Korner et al., 2021).

In this study, we investigated bacterial tick-borne diseases at seven sites in Germany. Nine to 10 years later, the same sites were re-investigated, and changes of the infection rates were discussed.

## 2 Materials and methods

### 2.1 Study area and tick collection

**Figure 1:**
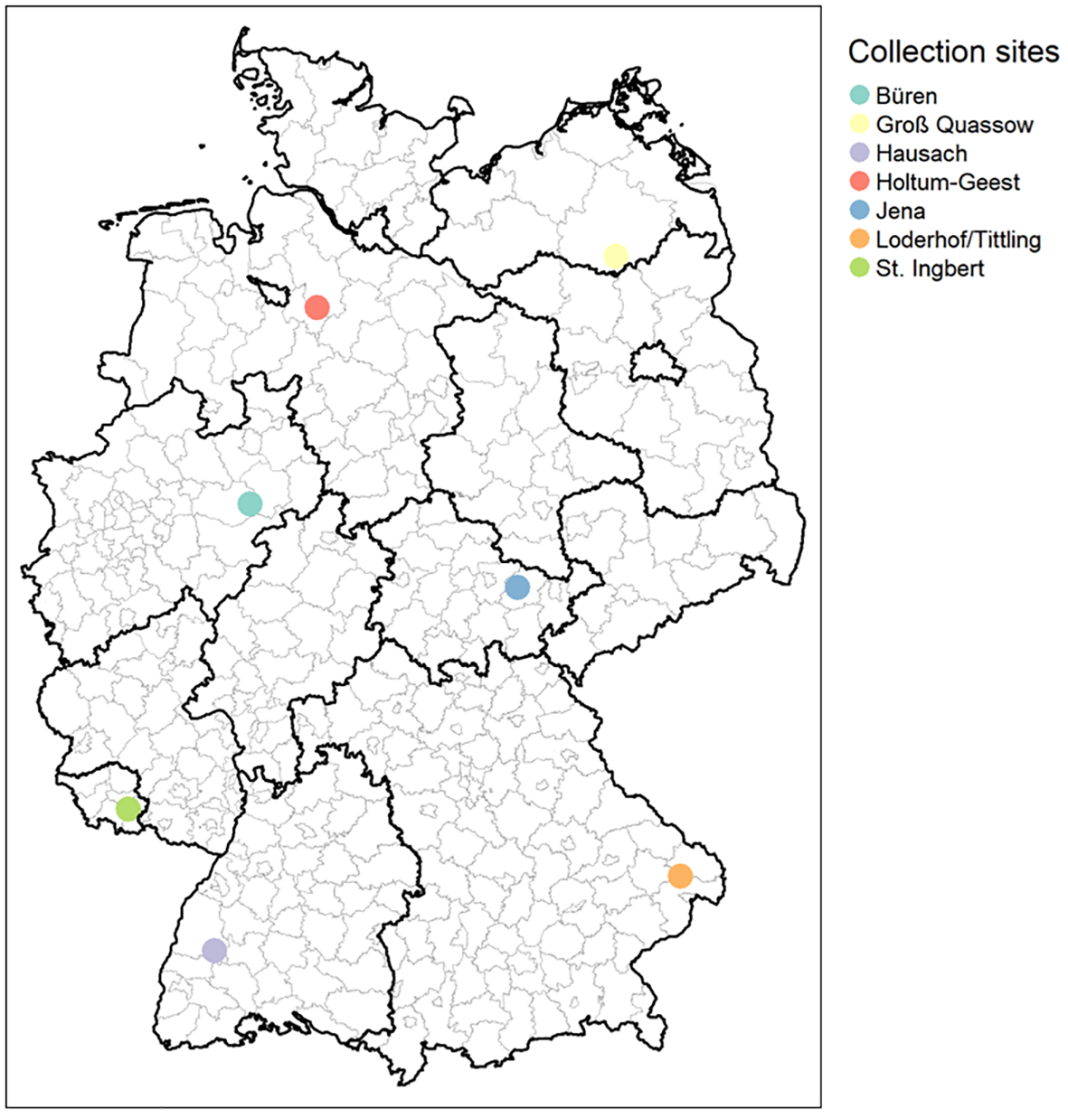
Geographical location of tick collection sites. Büren, North Rhine-Westphalia (NW); Groß Quassow, Mecklenburg-West Pomerania (MV); Hausch, Baden-Wuerttemberg (BW); Holtum-Geest, Lower Saxony (NI); Jena, Thuringia (TH); Loderhof/Tittling, Bavaria (BY); and St. Ingberg, Saarland (SL). Figure created with Maps ^©^ Mapbox (www.mapbox.com/about/maps) and ^©^ OpenStreetMap (www.openstreetmap.org/about).

Tick sampling was carried out at seven different sites located in seven German federal states from April until July in 2010 and in 2019, respectively. Only samples from Thuringia were collected from April until July in 2009 and 2019 (**Supplementary Table 1**; **Figure 1**). Ticks were collected by flagging with a cotton blanket of 1-m^2^ size in deciduous forest habitats with close proximity to hiking trails. After transfer to the laboratory, ticks were sorted as adult female or adult male ticks, nymphs, or larvae by morphological criteria. Species determination was conducted for all ticks according to Estrada-Peña et al. (2004).

### 2.2 DNA extraction

From all collection sites, a total of 1,608 *Ixodes ricinus* nymphs with an average of 115 nymphs per site and year were individually processed for DNA extraction using the NucleoSpin RNA/DNA kit (Macherey-Nagel, Düren, Germany) according to the manufacturer’s instructions. Briefly, ticks were ground individually in a mixer mill (Retsch, Haan, Germany) with three stainless steel beads in 350 of µl RA buffer for 2 min at 30 Hz. Tick debris were removed by centrifugation (11.000 x g, 1 min) and the lysate used for DNA extraction as indicated by the supplier’s instructions. All samples were stored at −80°C until further analysis.

### 2.3 Detection of *Borreliella* spp., *Borrelia* spp., and species differentiation

The ticks were examined for *Borreliella* spp. and *Borrelia* spp. DNA by real-time PCR targeting the 5S-23S intergenic spacer as described elsewhere (Strube et al., 2010). Detection of *Borrelia miyamotoi* was carried out by real-time PCR targeting the *flaB* gene as previously described (Venczel et al., 2016). Genospecies identification was carried out by *ospA* amplification and sequencing as described elsewhere (Rauter et al., 2002). Briefly, amplified *ospA* PCR fragments of 296 bp were separated by agarose gel electrophoresis and extracted (QIAquick Gel Extraction Kit, Qiagen, Hilden, Germany), and both strands were sequenced (Eurofins, Ebersberg, Germany). Raw data were trimmed with the Geneious Prime software (version 2021.0.1, Biomatters Ltd., Boston, USA), and data were analyzed using the online blastn tool (https://blast.ncbi.nlm.nih.gov/Blast.cgi) (Zhang et al., 2000). Samples used for species differentiation of *Borreliella* spp. and *Borrelia miyamotoi* could not be further investigated for other pathogens due to limited amount of DNA.

### 2.4 Detection of *Rickettsia* spp., *Anaplasma* spp., and *Coxiella burnetii*

Tick samples were screened by real-time quantitative PCR (qPCR) for *Rickettsia* spp. (*gltA*), *Anaplasma* spp. (16S rRNA), and *A. phagocytophilum* (*msp2*) and *C. burnetii* (IS1111), as described elsewhere (Courtney et al., 2004; Panning et al., 2008; Wolfel et al., 2008; Hurtado et al., 2015; Del Cerro et al., 2022). For species differentiation of rickettsiae, the *rrs*, *gltA*, and *ompB* genes were amplified and sequenced as described previously (Weisburg et al., 1991; Roux and Raoult, 2000; Labruna et al., 2004). Nucleotide sequences of single genes or concatemers were aligned to representative sequences of rickettsial species available from National Library of Medicine (NLM) GenBank (https://www.ncbi.nlm.nih.gov/genbank/) using MAFFT (v7.450) implemented in Geneious Prime. Alignments were manually trimmed and phylogenetic trees constructed using the neighbor-joining method with the genetic distance model Tamura-Nei and bootstrap tests with 1,000 replicates. *Ehrlichia chaffeensis* was set as outgroup for *rrs* and *gltA* and *R. bellii* for *ompB*. Phylogenetic trees were visualized using FigTree v1.4.3 (http://tree.bio.ed.ac.uk/software/figtree/).

### 2.5 Statistical analysis

To evaluate if there is a difference in the number of positive ticks between the Federal States (collection sites) and the two time points, we used the Fisher exact test (Fisher, 1922). All tests were carried out using the Statistical software R (R Core Team, 2021).

## 3 Results

### 3.1 Overall prevalence of tick-borne pathogens

The spatial and temporal distribution of tick-borne bacterial pathogens in Germany was examined. A total of 1,608 questing *I. ricinus* nymphs were collected from seven different locations representing seven federal states in 2009/2010 (n = 807) or 2019 (n = 801) (**Figure 1**). All nymphs were individually processed and screened by qPCR for bacterial pathogens.

**Figure 2:**
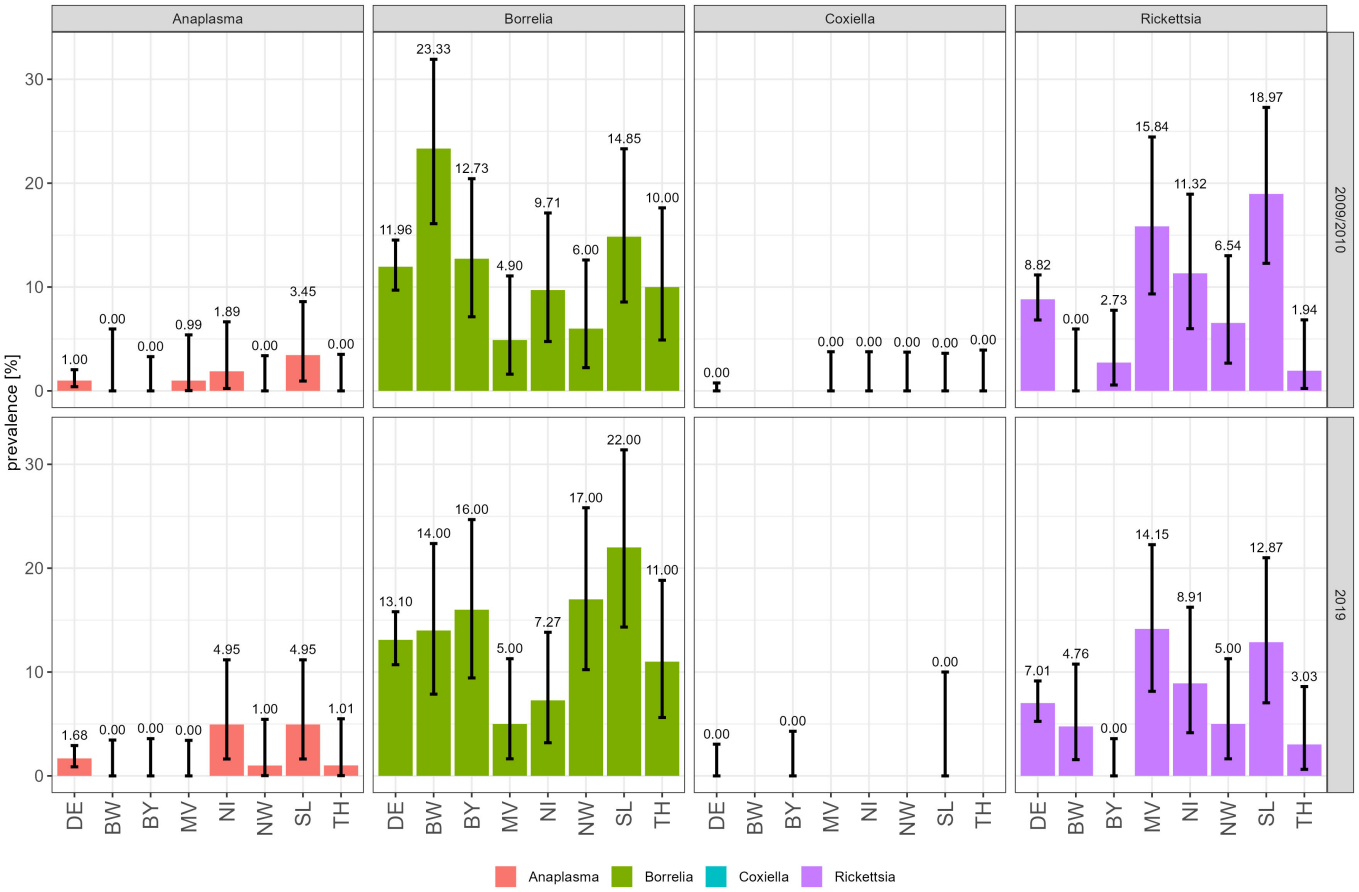
Prevalence of tick-borne pathogens in 2009/2010 (upper panel) and 2019 (lower panel) according to sampling site. Prevalence of *Anaplasma* spp. and *Borreliella* spp. including *B*. *miyamotoi*, *C*. *burnetii*, and *Rickettsia* spp. is indicated as bars with lower and higher confidence intervals. DE, Germany; BW, Baden-Wuerttemberg; BY, Bavaria; NI, Lower Saxony; MV, Mecklenburg-West Pomerania; NW, North Rhine-Westphalia; SL, Saarland; TH, Thuringia.

From all collected nymphs, 88/736 (11.96%) were positive for *Borreliella* spp., including *Borrelia miyamotoi*, 62/703 (8.82%) for *Rickettsia* spp., 7/703 (1%) for *Anaplasma* spp., and 0/424 (0%) for *Coxiella burnetii* in 2009 and 2010. No significant differences were observed for ticks collected in 2019 with 93/710 (13.10%) nymphs positive for *Borreliella* spp. including *Borrelia miyamotoi*, 50/708 (7.01%) for *Rickettsia* spp., 12/713 (1.68%) for *Anaplasma* spp., and 0/176 (0%) for *C. burnetii* (**Supplementary Table 1**). The overall results are summarized in **Figure 2**.

### 3.2 Detection of *Borreliella* spp. and *Borrelia miyamotoi*

For *Borreliella* spp., on average, 100 nymphs per collection site and year were screened (n = 1,446). The overall prevalence for *Borreliella* spp. including *Borrelia miyamotoi* in nymphs increased slightly from 11.96% (88/736) in 2009/2010 to 13.10% (93/710) in 2019 (**Figure 2**). However, there are some remarkable differences between the seven sites: In North Rhine-Westphalia (NW), *Borreliella* spp. including *Borrelia miyamotoi* prevalence in *I. ricinus* nymphs increased from 6.00% (6/100) in 2009/2010 to 17.00% (17/100) in 2019 and, in Saarland (SL), from 14.85% (15/101) to 22.00% (22/100). In Baden-Wurttemberg (BW), the prevalence decreased from 23.33% (28/120) in 2009/2010 to 14% (14/100) in 2019. At all other sites, the prevalence of *Borreliella* spp. including *Borrelia miyamotoi* remained nearly the same in 2009/2010 and 2019 (**Table 1**; **Figure 2**). Observed differences were statistically significant (Fisher test) in NW between 2009/2010 and 2019 only (**Table 2**).

**Table 1:** Prevalence of *Borreliella* spp. and *B. miyamotoi* in *Ixodes ricinus* nymphs in Germany in 2009/2010 compared to 2019.

	2009/2010		2019		Prevalence		Comparison 2009/2010 and 2019	
Federal state	Number of ticks analyzed	Positive ticks	Number of ticks analyzed	Positive ticks	2009/2010 (CI)	2019 (CI)	P-value	OR (CI)
BW	120	28	100	14	23.33 (16.10, 31.93)	14.00 (7.87, 22.37)	0.18	0,60 (0.28, 1.26)
BY	110	14	100	16	12.73 (7.14, 20.43)	16.00 (9.43, 24,68)	0.57	1,26 (0.54, 2.93)
NI	103	10	110	8	9.71 (4.76, 17.13)	7.27 (3.19, 13.83)	0.63	0,75 (0.25, 2,20)
MV	102	5	100	5	4.90 (1.61, 11.07)	5.00 (1.64, 11.28)	1.00	1,02 (0.23, 4.58)
NW	100	6	100	17	6.00 (2.23, 12.60)	17.00 (10.23, 25.82)	0.05	2,82 (1.01, 9.11)
SL	101	15	100	22	14.85 (8.56, 23.31)	22.00 (14.33, 31.39)	0.29	1,48 (0.69, 3.26)
TH	100	10	100	11	10.00 (4.90, 17.62)	11.00 (5.62, 18.83)	1.00	1,10 (0.40, 3.03)

CI, confidence interval; OR, odds ratio; BW, Baden-Wuerttemberg; BY, Bavaria; NI, Lower Saxony; MV, Mecklenburg-West Pomerania; NW, North Rhine-Westphalia; SL, Saarland; TH, Thuringia.

**Table 2:** Prevalence of *Rickettsia* spp. in *Ixodes ricinus* nymphs in Germany in 2009/2010 compared to 2019.

	2009/2010		2019		Prevalence		Comparison 2009/2010 and 2019	
Federal state	Number of ticks analyzed	Positive ticks	Number of ticks analyzed	Positive ticks	2009/2010 (CI)	2019 (CI)	P-value	OR (CI)
BW	60	0	100	5	0 (0, 5.96)	4.76 (1.56 10.76)	0.16	
BY	110	3	101	0	2.73 (0.57, 7,76)	0 (0.00, 3,59)	0.25	0 (0.00, 2.70
NI	106	12	101	9	11.32 (5.99, 18.94)	8.91 (4.16, 16.24)	0.65	0.79 (0.28, 2.14)
MV	101	16	106	15	15.84 (9.33, 24.45)	14.15 (8.14, 22.26)	0.85	0.89 (0.39, 2.04)
NW	107	7	100	5	6.54 (2.67, 13.02)	5.00 (1.64, 11.28)	0.77	0.77 (0.19, 2.90)
SL	116	22	101	13	18.97 (12.28, 27.29)	12.87 (7.04, 21.00)	0.36	0.68 (0.30, 1.49)
TH	103	2	99	3	1.94 (0.24, 6.84)	3.03 (0.63, 8.60)	0.68	1.56 (0.17, 19.01)

CI, confidence interval; OR, odds ratio; BW, Baden-Wuerttemberg; BY, Bavaria; NI, Lower Saxony; MV, Mecklenburg-West Pomerania; NW, North Rhine-Westphalia; SL, Saarland; TH, Thuringia.

**Figure 3:**
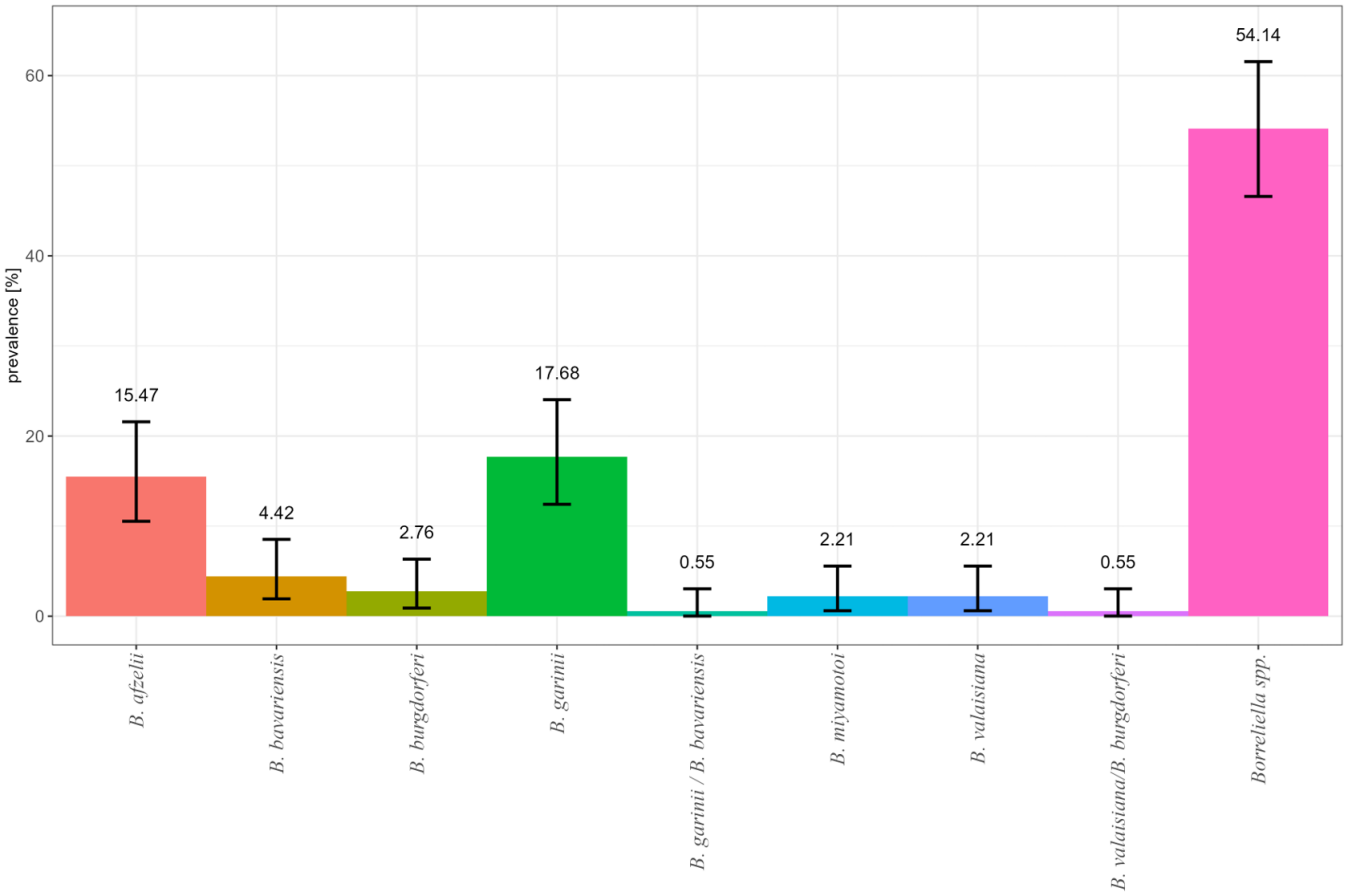
Relative frequency of *Borreliella* spp. and *Borrelia miyamotoi* in infected *I. ricinus* nymphs by real-time PCR detection in Germany in 2009/2010 and 2019.

Positive samples (n = 181) were subjected to species differentiation, whereof 57 resulted in no detectable PCR product and 38 were not further analyzed due to screening for other pathogens or poor DNA quality. In 79 samples, *B. garinii*, *B. afzelii*, *B. bavariensis*, *B. valaisiana*, *B. burgdorferi* s.s., and *Borrelia miyamotoi* were detected. The most frequently detected species were *B. afzelii* and *B. garinii* with 15.47% (n = 28) and 17.68% (n = 32), respectively. The other four species were detected remarkably less frequently in all *Borreliella* spp.–positive samples (**Figure 3**). Mixed infections of *Borrelia miyamotoi* and *B. afzelii* or *Borrelia miyamotoi* and *B. garinii* were detected in three ticks. For one tick, sample differentiation between *B. garinii* and *B. bavariensis* was not possible (**Figure 3**). Detailed information is presented in **Supplementary Figure 1**; **Supplementary Table 1**.

### 3.3 Detection of *Rickettsia* spp.

On average, 100 *I. ricinus* nymphs were tested for *Rickettsia* spp. per location and time point (n = 1,411), except for BW, 60 nymphs were tested in 2009/2010 only (**Table 2**; **Supplementary Table 1**). The overall prevalence in 2009/2010 was 8.82% (62/703) with a slight but statistically insignificant decrease to 7.06% (50/708) in 2019. Rickettsiae were most frequently detected in SL with 18.97% (22/116), in Mecklenburg-West Pomerania (MV) with 15.84% (16/101) and Lower Saxony (NI) with 11.32% (12/106) in 2009/2010. Similar results were obtained for samples taken in 2019 with 12.87% (13/101) positive ticks in SL, 14.15% (15/106) in MV, and 8.91% (9/101) in NI. In all other locations, none or ≤ 5 samples tested positive for *Rickettsia* spp. (**Table 1**).

**Figure 4:**
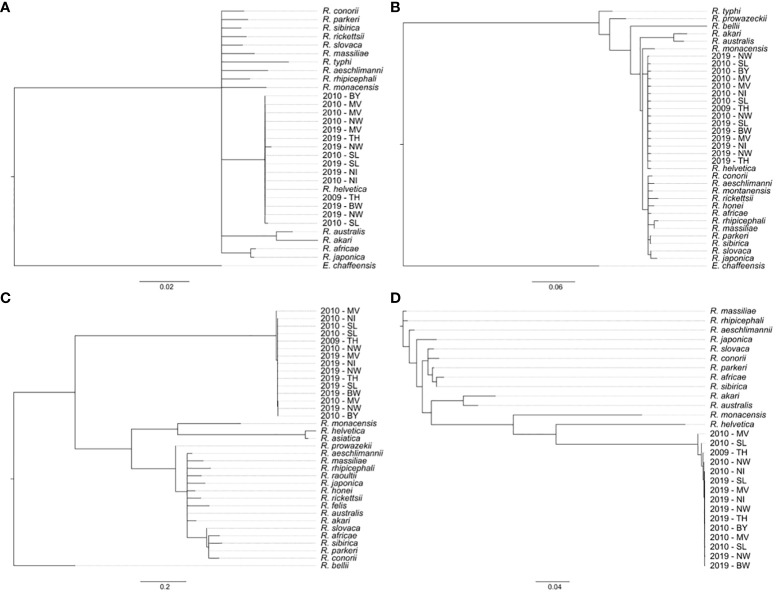
Phylogenetic tree based on **(A)** *rrs*, **(B)** *gltA*, and **(C)** *ompB* partial fragments or **(D)** *rrs*-*gltA*-*ompB*-concatemers from *Rickettsia* spp. reference strains and representative samples per sampling location and time. Trees were built using the neighbor-joining method with the genetic distance model Tamura-Nei and bootstrap tests with 1,000 replicates. Bar indicates nucleotide distance. Nucleotide accession numbers of *Rickettsia* spp. reference genes are listed in **Supplementary Table 2**; BW, Baden-Wuerttemberg; BY, Bavaria; NI, Lower Saxony; MV, Mecklenburg-West Pomerania; NW, North Rhine-Westphalia; SL, Saarland; TH, Thuringia.

For species differentiation, all 112 *Rickettsia* spp.–positive samples were subjected to PCR amplification and sequencing of *rrs*, *gltA*, and *ompB* gene fragments. Overall, from 100, 88, and 103 samples, a *rrs* gene fragment, a *gltA* gene fragment, and an *ompB* gene fragment were generated, respectively. All three gene fragments were obtained from 79 samples. All retrieved sequences from the *rrs* and *gltA* gene fragments were identical (**Figures 4A, B**). Four sequence types of *ompB* (**Figure 4C**) were observed and six sequence types when all three gene fragments were aligned as concatemers (**Figure 4D**). All positive (108/112) samples clustered together with spotted fever (SFG) group rickettsiae and were identified as *R. helvetica*.

### 3.4 Detection of *Anaplasma* spp.

*Anaplasma* were detected in 19 out of 1,416 tested *I. ricinus* nymphs (**Table 3**). There was no statistically significant difference between sampling sites and time with 1.00% (7/703) in 2009/2010 and 1.68% (12/713) in 2019. Most *Anaplasma* positive ticks were found in SL and NI with a slight increase in prevalence from 3.45% (4/116) to 4.95% (5/101) or 1.89% (2/106) to 4.95% (5/101). Of these, seven [0.469% (0.200, 1.021)] tested positive for *A. phagocytophilum* collected in MV in 2010 (n = 1), in NI in 2019 (n = 1), in SL in 2010 (n = 3), and 2019 (n = 2).

**Table 3:** Prevalence of *Anaplasma* spp. in *Ixodes ricinus* nymphs in Germany in 2009/2010 compared to 2019.

	2009/2010		2019		Prevalence		Comparison 2009/2010 and 2019	
Federal state	Number of ticks analyzed	Positive ticks	Number of ticks analyzed	Positive ticks	2009/2010 (CI)	2019 (CI)	P-value	OR (CI)
BW	60	0	105	0	0 (0.00, 5.96)	0 (0.00, 3.45)	1.00	
BY	110	0	101	0	0 (0.00, 3.30)	0 (0.00, 3.59)	1.00	
NI	106	2	101	5	1.89 (0.23, 6.65)	4.95 (1.63, 11.18)	0.28	2.61 (0.42, 28.03)
MV	101	1	106	0	0.99 (0.03, 5.39)	0 (0.00, 3.42)	0.49	0 (0.00, 37.53)
NW	107	0	100	1	0 (0.00, 3.39)	1.00 (0.03, 5.45)	0.49	
SL	116	4	101	5	3.45 (0.95, 8.59)	4.95 (1.63, 11.18)	0.74	1.43 (0.30, 7.43)
TH	103	0	99	1	0 (0.00, 3.52)	1.01 (0.03, 5.50)	0.49	

CI, confidence interval; OR, odds ratio; BW, Baden-Wuerttemberg; BY, Bavaria; NI, Lower Saxony; MV, Mecklenburg-West Pomerania; NW, North Rhine-Westphalia; SL, Saarland; TH, Thuringia.

### 3.5 Detection of *Coxiella burnetii*

Nearly 600 *I. ricinus* nymphs collected in 2009/2010 (n = 424) from five different locations and in 2019 (n = 176) from two different locations were analyzed for *C. burnetii*. All samples tested negative for the IS1111 element. Because of the overall negative results, further testing of samples was neglected.

## 4 Discussion

Tick-borne diseases are of high interest in human and veterinary medicine. Observations about their spread in space and time can help to assess the epidemiological situation in a given area. The vector *I. ricinus* has a broad range of host species including mammals, birds, and reptiles. The compilation of a high-resolution density map of unfed nymphal *I. ricinus* allows an insight into the broad distribution of this important disease vector in Germany depending on bioclimatic variables and land cover (Brugger et al., 2016). In our study, the prevalence for *Borreliella* spp. including *Borrelia miyamotoi* in nymphal *I. ricinus* was 11.96% in 2009/2010 and 13.10% 10 years later (**Table 1**). This is in accordance with many other studies, e.g., with prevalence of 10%, between 5% and 12% or 12.9% in nymphs (Wilske et al., 1987; Kampen et al., 2004; Fingerle et al., 2008). A higher prevalence in nymphs was found in South Germany (15%) by Raileanu et al. (2021), in Hanover in Northwest Germany (19.8%) by Blazejak et al. (2018) and in North Germany (28.6%) by Knoll et al. (2021a) with prevalence between 18.6% and 45.7% in nymphs at different places and habitats. Prevalence for *B. burgdorferi* s.l. in Europe was reported between 2% and 43% for nymphs, with a mean average of 10.8% (Hubalek and Halouzka, 1998). A meta-analysis of studies in Europe between 2010 and 2016 showed an average *Borreliella* prevalence of 15.6% (Strnad et al., 2017).

Comparable results were shown by Kampen et al. (2004) at three sites in the region Siebengebirge in West Germany. They found a higher prevalence of 5.5%, 15.8%, and 21.8% at these locations in 2001 compared to 1987–1989 with 1.1% to 15.4% (Kurtenbach et al., 1995; Kampen et al., 2004). This change might be caused by presently unknown changes, e.g., in ecological conditions or wildlife management. Schwarz et al. (2012) detected an increasing *B. burgdorferi* s.l. prevalence in the same region over the last two decades (Schwarz et al., 2012). In our study, only at one site, a significant higher prevalence was observed (NW) after 10 years.

The detected differences may be caused by changes in ecological conditions or wildlife management, like it was assumed by Kampen et al. (2004) for three sites in the Siebengebirge in West Germany. In Hanover in Northwest Germany, it was assumed that the reason for decreased *B. afzelii* detection over 10 years could be caused by changed reservoir host population (Blazejak et al., 2018). It is suggested that the bird population could be responsible for local changes of *Borreliella* spp. prevalence like in the here presented study (Klaus et al., 2016). Birds, especially blackbirds (*Turdus merula*) and song thrushes (*Turdus philomelos*), play an important role in the distribution of *Borreliella* spp (Humair et al., 1993; Olsen et al., 1995; Taragel’ova et al., 2008; Klaus et al., 2016). They can also serve as reservoir hosts (Lommano et al., 2014), but there are no data available about bird populations at the sampling sites visited in the here presented study. The population size of abundant and common bird species increased generally from 2009 until 2018, but trends are different according to habitat and nest sides (Kamp et al., 2021). The observed increase of overall prevalence of *Borreliella* spp. might reflect the general increase of birds as tick hosts, but this is only speculative.

In our study, in 2009/2010, the dominant species was rodent-associated *B. afzelii* with up to 40% in SL and TH whose prevalence declined to 27.27% or 9.09% in 2019 (**Supplementary Figure S1**). The same high percentage (43.1%) for *B. afzelii* was found in Bavaria in 2010 (Vogerl et al., 2012). In 2019, the bird-associated species *B. garinii* increased at all sampling sites except BW and became the dominant species (**Supplementary Figure S1**). The reason is not quite clear. It is possible that such changes can be introduced by birds as transportation tool for ticks and reservoir hosts for *B. burgdorferi* s.l. species. A shift in *B. burgdorferi* s.l. species over a time span was observed not only by Blazejak et al. (2018) in Hanover, Germany, but also by Okeyo et al. (2020) during a longitudinal study between 1999 and 2010 in Latvia in one of three investigated habitats. In a former study, in Germany, it became evident that not only sedentary birds like blackbirds (*Turdus merula*) were the most frequently tick infested species, but also short- and long-distance bird species were more suitable to transfer *Borreliella* spp. strains to new sites (Klaus et al., 2016). However, the rodent-associated species *B. afzelii* can be harbored in ticks infesting birds and can be transported in this way as well (Olsen et al., 1995; Geller et al., 2013; Klaus et al., 2016). A study of Norte et al. (2020) in 11 European countries showed that 28 bird species transported three tick species. Among the ticks, 37% were infected with various *B. burgdorferi* s.l. species, especially bird-associated *B. garinii* (61%), but, among the species, also rodent-associated *B. afzelii* (9%). In our study, other *B. burgdorferi* s.l. species were found very rarely, i.e., *B. bavariensis* (4.42%), *B. valaisiana* (0.55%), and *B. burgdorferi* s.s. (2.76%) (**Figure 3**). Other studies showed higher prevalence for *Borreliella* spp., e.g., 9.7% for *B. valaisiana* and 9.9% *B. burgdorferi* s.s. in *I. ricinus* collected in northern Germany or 14.7% for *B. valaisiana* and 6.3% for *B. burgdorferi* s.s. in *I. ricinus* collected in southern Germany, respectively (Vögerl et al., 2012; Tappe et al., 2014). These studies analyzed all tick developmental stages, and the overall prevalence was reported. This might reflect that tick developmental stages differ in *Borreliella* spp. prevalence and might explain the here observed lower prevalence in nymphs.

A special remark should be made about *Borrelia miyamotoi* (2.21%) that was found in NW and SL in 2009/2010 and in Lower Saxony and Thuringia in 2019. Reports show that this species is established in Germany although at a very low level of 1.2% to 3.5% prevalence in *I. ricinus* ticks (Richter et al., 2003; Sinski et al., 2016; Szekeres et al., 2017). The clinical relevance of this *Borrelia* species should not be underestimated (Cutler et al., 2019; Kubiak et al., 2021). Clinical reports are rare but severe with central nervous system infections as chronic manifestations in immunocompromised persons (Gugliotta et al., 2013; Hovius et al., 2013; Boden et al., 2016). An association between the history of non-Hodgkin lymphoma and rituximab treatment, which causes conditional immunosuppression, was made in these patients. However, serological assays for lyme disease fail to detect *B. miyamotoi* infections and clinicians should rely on patient’s history, clinical examination, and routine cerebrospinal fluid (CSF) analysis. Classical dark-field microscopy or acrinidine orange staining and 16S RNA sequencing of CSF supports detection of spirochetes and *B. miyamotoi* (Boden et al., 2016).

*Rickettsiae* are distributed worldwide and cause mild to severe, sometimes life-threatening diseases in humans. Several *Rickettsia* spp. have been reported in Germany, but there is limited information available on their distribution and genetic diversity. Almost all *Rickettsia* spp. positive tick samples could be identified as *R. helvetica* (108/112 samples) in the here reported study. This species was discovered in 1979 in Switzerland and has since been reported in Germany in *I. ricinus* ticks with a prevalence up to 13.4% and in small rodents with up to 33.3% (Burgdorfer et al., 1979; Silaghi et al., 2011; Obiegala et al., 2016). The highest prevalence of *Rickettsia* spp. in *I. ricinus* ticks was recorded for the area of Hamburg in North Germany with 52.5%. Of all positive samples 25.6% were subjected to species differentiation with *R. helvetica* as the only occurring species (May and Strube, 2014). Other studies demonstrated a prevalence of 12% for *Rickettsia* spp. in *I. ricinus* and exclusively detected *R. helvetica* in South Germany (Wolfel et al., 2006). Monitoring of ticks for *Rickettsia* spp. over a 15-year period in Northern Germany in the region of Hannover showed significant fluctuations between sampling years, with a general increase from 2005 with 33.3% until 2020 with 36.0% and a peak of 50.8% in 2015 (Glass et al., 2022). This demonstrates, that the here reported prevalence from approximately 2% to 19% for some federal states and an overall prevalence of 8.82% for 2009/2010 or 7.01% for 2019 for *Rickettsia* spp. and the exclusively detected species *R. helvetica* in *I. ricinus* is comparable with former studies.

Albeit *I. ricinus* is considered as the main vector for *R. helvetica*, other rickettsiae, e.g., the highly pathogenic *R. conorii* have been detected in this vector (Sprong et al., 2009). Interestingly, *R. helvetica* was also detected in a botfly larvae from a roe deer questioning the animal reservoir (Scheid et al., 2016). It is assumed that small mammals function as animal reservoir for *R. helvetica*. Several studies show a high positivity of wild and companion animals by DNA detection or serological examination. Particulary, rodents and shrews carry *R. helvetica* as dominant occurring species and less frequent *R. felis* and *R raoultii*. The prevalence ranged from 6.8% in MV, 7.0% in NW, and 8.0% in TH (Fischer et al., 2018). Dogs as companion animals show a high seroprevalence of 93.9% for *Rickettsia* spp. antibodies using a micro-immunofluorescence assay. These dogs were not imported or have left Germany. Of all serological positive samples, 66.0% could be determined as specific for *R. helvetica*, 2.8% for *R. raoulti*, and 1.6% for *R. slovaca* (Wachter et al., 2015). Companion animals are in close contact with their owners and living area. Therefore, the pathogenic potential of *R. helvetica* and other *Rickettsia* spp. present in Germany and neighboring countries should not be underestimated. New tick species have been reported in Germany, such as *Hyalomma* spp. carrying the human pathogenic species *R. slovaca* and *R. aeschlimanii* (Chitimia-Dobler et al., 2019, 2024). Also, migrating birds carry ticks and their pathogenic cargo. One study discovered in addition to *R. helvetica* another species *Candidatus* R. vini in *I. arboricola* and *I. lividus* ticks for the first time in Germany. This species is closely related to the highly human pathogenic species *R. japonica* and *R. heilongjiangensis* (Wimbauer et al., 2022).

The prevalence of *Rickettsia* spp. and *Anaplasma* spp. is often analyzed and reported together. The here detected prevalence for *Anaplasma* spp. from approximately 2% to 5% for some federal states or 1.00 and 1.68% for 2009/2010 and 2019, respectively, is comparable to previously reported prevalence in *I. ricinus* ticks of 1.9% to 6.4% in different parts of Germany (Hartelt et al., 2004; Hildebrandt et al., 2010; Tappe and Strube, 2013; Knoll et al., 2021b). Differences in prevalence may be caused by variable geographical host densities, e.g., for wild ruminants and rodents (De la Fuente et al., 2005; Stuen et al., 2013).

No significant changes were observed between 2009/2010 and 2019. Of all *Anaplasma* positive ticks seven nymphs were positive for the zoonotic pathogen *A. phagocytophilum*. Reported co-infection rates of *A. phagocytophilum* and *Rickettsia* spp. are low with approximately 1% (Hildebrandt et al., 2010; Tappe and Strube, 2013). Co-infection was detected in two *I. ricinus* nymphs from 2010 or 2019 in NI only. However, *A. phagocytophilum* is an important pathogen affecting humans as well as companion animals like horses, cats, and dogs (Kohn et al., 2011; Langenwalder et al., 2020; Schafer et al., 2022a, b, 2023).

Only a few studies compare prevalence of tick-borne pathogens over time with contradictory results. Reports from the region of Hannover in northern Germany detected statistically significant increase for *A. phagocytophilum* and *Rickettsia* spp. positive ticks in 2010 compared to 2005 (Tappe and Strube, 2013). In contrast, a significant decrease in prevalence was detected in north-west Germany in 2019 compared to 2018 (Knoll et al., 2021b). Another study reported a stagnating prevalence for *A. phagocytophilum* but a significant increase for R*ickettsia* spp. in *I. ricinus* over a 10-year monitoring period (Blazejak et al., 2017). These data are comparable with the here reported prevalence for these pathogens with no significant changes after a decade.

Comprehensive studies on the prevalence of rickettsial diseases or HGA in Germany are missing. There are a few reports available showing a high seroprevalence for *Rickettsia* spp. in forestry workers or homeless people, which are more likely to be exposed to arthropods. Among homeless people 7% (10/147) tested serological positive for *R. conorii*, whereof only two originated not from Germany or neighboring countries (Heinrich et al., 2023). Further forestry workers show a high seroprevalence for SFG rickettsiae of 27.5% with 9.7% for *R. helvetica*, 5% *R. raoultii*, 2.7% *R. felis*, 0.5% *R. monacensis*, and 0.5% *R. slovaca* (Wolfel et al., 2017). Similar, the seroprevalence for *A. phagocytophilum* in forestry workers with previous tick exposure was reported as 4.51% and 1.20% in the control group (Kowalski et al., 2006). Reports on clinical cases are rare. This suggests that rickettsial diseases and HGA might be rare events in Germany or underdiagnosed.

Historically, Q fever in humans and Coxiellosis in ruminants was associated with a high abundance of the sheep tick *D. marginatus* in southern Germany. It was assumed that *C. burnetii* is either transmitted by the tick bite or excreted and spread by tick feces (Liebisch, 1977). Vector competence for two in Germany most abundant tick species in Germany, *I. ricinus* and *D. marginatus*, was shown recently. Both tick species can take up the bacteria with the blood meal and excrete them with feces. Transstadial transmission from nymphs to adult ticks was demonstrated but transovarial transmission seems unlikely (Korner et al., 2020; Bauer et al., 2023). However, several studies reporting an overall negative or very low prevalence for *C. burnetii* in ticks in Germany. A few reports are available targeting *Dermacentor* spp. ticks and small rodents in Q fever outbreak areas in southern Germany, but none of the tested samples were *Coxiella*-positive by PCR (Hartelt et al., 2008; Pluta et al., 2010). A small-scale study carried out in western Germany detected no *C. burnetii*–positive *I. ricinus* ticks (n = 52) (Henning et al., 2006). Only one study reported a small portion of *C. burnetii–*positive *I. ricinus* (1.9%) for a region in East Germany (Hildebrandt et al., 2011). Therefore, the prevalence of *C. burnetii* in *I. ricinus* and *Dermacentor* spp. ticks is generally assumed to be very low or negative in Germany and is in accordance with the here reported data.

The prevalence of *C. burnetii* in ticks increases in eastern and southern European countries, e.g., high prevalence for *C. burnetii* in *D. marginatus* from Serbia (22%) (Tomanovic et al., 2013) or France (2% to 13%) (Bonnet et al., 2013; Michelet et al., 2014) was reported. Prevalence of *C. burnetii* in *I. ricinus* varies with approximately 16% in Poland (Szymańska-Czerwińska et al., 2013) and negative results for Spain (Astobiza et al., 2011) or France (Michelet et al., 2014). This implies that foci in which ticks may play a role in transmission of *C. burnetii* do exist. However, all results need to be evaluated with care, because most studies do not employ methods for differentiation between *C. burnetii* and CLEs. The abundance of certain tick species seems to play a role in the spread of pathogens. High prevalence of *C. burnetii* in species of the genera *Hyalomma* spp. and *Rhipicephalus* spp. with up to 54% in Spain or 22% in Italy was reported (Mancini et al., 2014; Gonzalez et al., 2020), and vector competence was shown for *H. aegypticum* (Široký et al., 2010). Climate change might influence the distribution of tick species and *Hyalomma* spp. was already detected and shown to develop from nymphs to adult ticks in Germany (Chitimia-Dobler et al., 2019, 2024). However, *Hyalomma* ticks (n = 18) collected in Germany and examined for tick-borne pathogen were negative for *C. burnetii*, but the number of ticks examined was very low and may not be representative (Chitimia-Dobler et al., 2019).

## 5 Conclusions

Considering the high importance of bacterial tick-borne infections for human health, it is recommended to examine changes of pathogens in ticks as vector in space and time. Especially, longitudinal studies at the same places are rare because of high costs and technical problems. Here, we gave an insight at seven sites in Germany 9 to 10 years after the first examination with very different aspects at the single sites but no general increase of tick-borne pathogens during this time span. This may be caused by the multifactorial conditions at the sites, e.g., climate conditions and especially host population. These factors were not monitored in the here presented study but are necessary to understand which factors influence the prevalence of tick-borne pathogens, tick density, and transmission dynamics. Longitudinal observations are recommended by including such parameters like host species and density to improve our understanding of tick-borne diseases.

## Data availability statement

The datasets presented in this study can be found in online repositories. The names of the repository/repositories and accession number(s) can be found in the article/**Supplementary Material**.

## Ethics statement

The manuscript presents research on animals that do not require ethical approval for their study.

## Author contributions

KM: Data curation, Formal Analysis, Investigation, Methodology, Project administration, Resources, Supervision, Validation, Writing – original draft, Writing – review & editing. BH: Formal Analysis, Investigation, Resources, Writing – review & editing. JG: Data curation, Formal Analysis, Investigation, Resources, Validation, Visualization, Writing – review & editing. HB: Formal Analysis, Writing – review & editing. MP: Writing – review & editing. CK: Conceptualization, Data curation, Formal Analysis, Investigation, Methodology, Project administration, Resources, Supervision, Validation, Writing – original draft, Writing – review & editing.
