# Supplementary material for: Prevalence of tick-borne bacterial pathogens in Germany—has the situation changed after a decade?
Source: Front Cell Infect Microbiol. 2024 Jul 18;14:1429667. doi: 10.3389/fcimb.2024.1429667 (PMC11291221; doi:10.3389/fcimb.2024.1429667)
Supplement: Supplementary file 2 [file DataSheet_1.docx]

**Supplementary Information**

Table 2: *Rickettsia* spp. and *Ehrlichia* spp. reference genes used for phylogenetic analysis

| Species | *GenBank Accession no.* | | |
| --- | --- | --- | --- |
|  | ***gltA*** | ***ompB*** | ***rrs*** |
| *E. chaffeensis* | AF304142 | - | AF147752 |
| *R. aeschlimanni* | RSU59722 | AF123705 | RAU74757 |
| *R. africae* | RAU59733 | AF123706 | RIRRGDA |
| *R. akari* | RAU59717 | AF123707 | RIRRGDB |
| *R. asiatica* | - | DQ110870 | - |
| *R. australis* | RAU59718 | AF123709 | RIRRGDD |
| *R. bellii* | RBU59716 | AY970508 | - |
| *R. conorii* | AE008677 | AF123721 | AF541999 |
| *R. felis* | - | AF182279 | - |
| *R. helvetica* | RHU59723 | AF123725 | RIRRGDK |
| *R. honei* | AF022817 | AF123724 | - |
| *R. japonica* | RJU59724 | AF123713 | RIRRGDL |
| *R. massiliae* | RMU59719 | AF123714 | RIRRGDM |
| *R. monacensis* | DQ100163 | EF380356 | NR_115686 |
| *R. montanensis* | RMU74756 | - | - |
| *R. parkeri* | RPU59732 | AF123717 | RIRRRDA |
| *R. prowazeckii* | NC_000963 | AF1237189 | - |
| *R. raoultii* | - | EU036984 | - |
| *R. rhipicephali* | RRU59721 | AF123719 | RIRRGDO |
| *R. rickettsii* | RRU59729 | X16353 | RIRRGDP |
| *R. sibirica* | RSU59734 | AF123722 | RIRRGDR |
| *R. slovaca* | RSU59725 | AF123723 | RIRRGDX |
| *R. typhi* | RTU59714 | - | RIRRGDU |


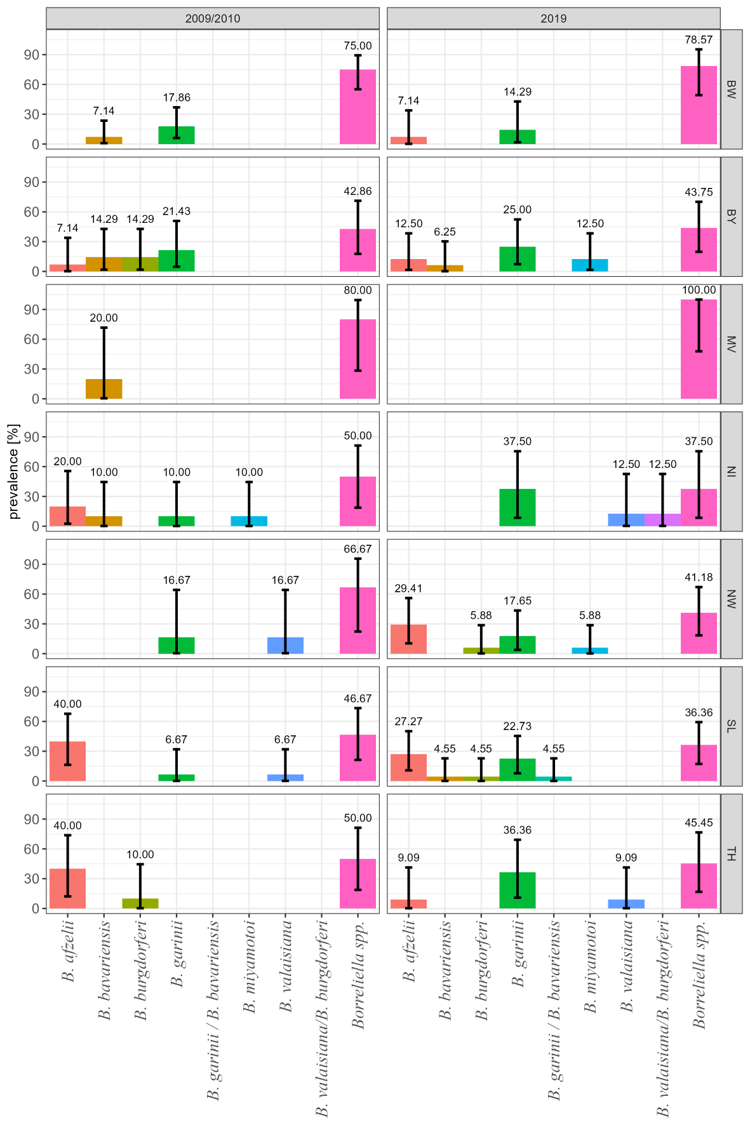


Figure S1: Prevalence of *Borreliella spp*. and *Borrelia miyamotoi* in *Ixodes ricinus* nymphs in 2009/2010 and 2019 in seven federal states of Germany. Ticks were sampled by flagging and individual processed for DNA isolation. *Borreliella* spp. and *Borrelia miyamotoi* were detected using PCR and Sequencing as described elsewhere [1-3].

References

1. Rauter, C.; Oehme, R.; Diterich, I.; Engele, M.; Hartung, T. Distribution of clinically relevant *Borrelia* genospecies in ticks assessed by a novel, single-run, real-time PCR. *J Clin Microbiol* **2002**, *40*, 36-43, doi:10.1128/JCM.40.1.36-43.2002.

2. Strube, C.; Montenegro, V.M.; Epe, C.; Eckelt, E.; Schnieder, T. Establishment of a minor groove binder-probe based quantitative real time PCR to detect *Borrelia burgdorferi* sensu lato and differentiation of *Borrelia spielmanii* by *ospA*-specific conventional PCR. *Parasit Vectors* **2010**, *3*, 69, doi:10.1186/1756-3305-3-69.

3. Venczel, R.; Knoke, L.; Pavlovic, M.; Dzaferovic, E.; Vaculova, T.; Silaghi, C.; Overzier, E.; Konrad, R.; Kolencik, S.; Derdakova, M.; et al. A novel duplex real-time PCR permits simultaneous detection and differentiation of *Borrelia miyamotoi* and *Borrelia burgdorferi* sensu lato. *Infection* **2016**, *44*, 47-55, doi:10.1007/s15010-015-0820-8.
